# Ashwagandha Does Not Enhance the Effect of High-Intensity Interval Training on Selected Energy Metabolism Parameters in Young Healthy Men

**DOI:** 10.3390/nu17203245

**Published:** 2025-10-16

**Authors:** Małgorzata Charmas, Ewa Jówko, Barbara Długołęcka, Andrzej Klusiewicz, Iwona Przybylska, Anna Galczak-Kondraciuk

**Affiliations:** 1 Department of Physiology and Biochemistry, Faculty of Physical Education and Health in Biała Podlaska, University of Physical Education in Warsaw, 00-968 Warsaw, Poland; ewa.jowko@awf.edu.pl (E.J.); barbara.dlugolecka@awf.edu.pl (B.D.); andrzej.klusiewicz@awf.edu.pl (A.K.); 2 Regional Research and Development Centre, Faculty of Physical Education and Health in Biała Podlaska, University of Physical Education in Warsaw, 00-968 Warsaw, Poland; iwona.przybylska@awf.edu.pl; 3 Department of Health Promotion, Faculty of Physical Education and Health in Biała Podlaska, University of Physical Education in Warsaw, 00-968 Warsaw, Poland; anna.kondraciuk@awf.edu.pl

**Keywords:** high-intensity interval training, ashwagandha supplementation, body composition, lipid profile, adiponectin, asprosin, irisin

## Abstract

Background/Objectives: High-intensity interval training (HIIT) is considered an effective way in improving aerobic capacity and selected health parameters. Ashwagandha is an herb with possible health-promoting properties that may affect metabolism and performance. The aim of this study was to evaluate the effects of ashwagandha supplementation (600 mg/day) during an 8-week HIIT on body composition, lipid profile and hormone levels related to energy homeostasis in healthy young men. Methods: The study was randomised, double-blind and placebo-controlled (Placebo group, PL, *n* = 20; ashwagandha, A, *n* = 18). HIIT was conducted on a rowing ergometer (3 times per week, 5–7 series of 1.5 min at 85–95% of maximum power, with intervals of 1.5 min at 70 W). Body composition (BIA, Tanita TBF 300P), serum lipid profile (tChol, HDL-cholesterol, LDL-cholesterol, TG) and serum levels of adiponectin, asprosin and irisin were analysed before (term 1) and after the8-week study (term 2). Both the lipid and hormonal profiles were measured in three time points: pre- and post-graded exercise test and after 24 h recovery period. Results: Analysis showed no effect of training or supplementation on body composition and lipid profile (*p* > 0.05). In turn, the 8-week HIIT decreased resting levels of adiponectin and increased irisin levels post-exercise and after 24 h (*p* < 0.05). Conclusions: In young, healthy men, an 8-week HIIT programme significantly affects selected hormones related to energy metabolism of adipose (adiponectin) and muscle (irisin) tissues, but ashwagandha supplementation did not significantly affect any of the hormonal parameters analysed.

## 1. Introduction

High-intensity interval training (HIIT) is an exercise strategy characterised by alternating periods of intense activity at different durations and low-intensity/passive recovery periods. It is generally used to improve cardiovascular function and increase metabolic rate [1,2,3,4,5]. High-intensity interval training (HIIT) can significantly affect the body’s physiology and overall health, offering many benefits over other types of physical activity undertaken by different groups of people, often in a more time-efficient manner than, for example, moderate-intensity continuous training. The available scientific literature shows the impact of HIIT on many aspects of the human body starting from metabolic activity to aspects as practical as the time spent in physical activity, which has been shown to be a very important reason for many people to undertake any activity [6,7].

High-intensity interval training (HIIT) has beneficial effects in improving body shape and body composition in obese individuals and has been shown to be effective in reducing body fat percentage [1,8,9] and improving biochemical markers. Meta-analyses show that in overweight and obese individuals, HIIT leads to significant reductions in whole-body fat mass and waist circumference [8] and achieves these results with approximately 40% less training time [1]. Other studies also suggest that HIIT may be particularly effective in reducing visceral fat [8,10]. In contrast, research by Hov et al. has shown that high-intensity interval training should be implemented as part of standard clinical care in patients with non-specific musculoskeletal conditions because of the positive association between improved physical performance and physical and emotional functioning [11].

A number of beneficial physiological effects of HIIT within the cardiovascular system have been identified, both acutely and chronically. Even a single HIIT session can lead to transient vasodilation and reduced arterial stiffness, which is associated with improved haemodynamics and endothelial function [4]. Regular use of HIIT protocols can contribute to increased aerobic capacity, improved cardiac minute volume and autonomic regulation [3]. In individuals with reduced physical activity and excessive body weight, HIIT can lead to significant improvements in cardiometabolic indices and weight reduction, with a concomitant increase in cardiovascular efficiency [1,3]. Importantly, these effects often occur with a shorter total training duration compared to classic continuous moderate-intensity exercise [1,5].

This article describes two intervention factors, i.e., high-intensity interval exercise and ashwagandha supplementation. Ashwagandha (*Withania somnifera* L.), also known as Indian ginseng or sluggish withania, is one of the key plants used in Ayurveda for more than 3000 years, where it plays the role of Rasayana, i.e., supporting physical and mental well-being. Reviewing information in publicly available online sources, one can find that this herb is valued for its broad spectrum of health-promoting properties, including adaptogenic effects. The chemical composition of this plant includes about 35 identified active substances, the most important of which are withanolides and alkaloids, while flavonoids, saponins, coumarins, phytosterols, nitrogenous compounds, resins, lipids, carbohydrates and fatty acids are also present. Withanolides, such as withapherin A or withanolides A-Y, are mainly found in the leaves, while acylated sterol glycosides and glycowithanolides are present in the root [12]. A review of clinical and experimental studies confirms the pleiotropic effects of ashwagandha [13,14]. It increases the body’s resistance to stress not only in rodents [15,16], but also in humans, including calming and tonic effects reducing anxiety and depressive symptoms [17,18,19], as well as improving cognitive function and exhibiting neuroprotective properties [13,14,20]. Additionally, it has been observed that ashwagandha supplementation can contribute to increased physical performance [14,20,21,22], muscle regeneration and strength [21,22], as well as increased testosterone [13] and reduced cortisol levels [13,19]. Furthermore, ashwagandha is indicated to have a potentially beneficial effect on metabolic health, showing antidiabetic [13,23,24], cardioprotective [25] and immunomodulatory properties [13,14,20] as well as normalising thyroid hormone levels [13,26]. Systematic reviews have shown that ashwagandha supplementation can improve weight loss and have a beneficial effect on blood glucose levels and lipid profile. The exact metabolic pathways underlying these effects are not yet well understood, but as indicated, interactions between inflammatory factors and hormones cannot be ruled out [27]. Overall, the available clinical studies support the efficacy and safety of ashwagandha as a resource for supporting mental and physical health, although many of the scientific reports by the authors emphasise the need for further research. It should be noted that the vast majority of these reports are based on results obtained in the Indian population. It is worth noting, however, that the available scientific literature does not provide answers to the question of what effect ashwagandha extract has on healthy people without chronic diseases and the effectiveness of the physical activities they undertake.

It has been indicated that the health-promoting effects of ashwagandha are dose-dependent and influenced by the duration of supplementation and the standardisation of the extract. Clinical trials have used a wide range of doses, from low (120–250 mg/day) to moderate and high doses (500–1000 mg/day) of standardised root extract [12,17,18,19,21]. Higher doses, typically around 500–600 mg/day, administered over 6–8 weeks, are most consistently associated with improvements in stress and mood parameters, enhanced sleep quality, and better metabolic profiles [17,18,19,23,24,25]. Moderate doses (300–500 mg/day) have been linked to improved lipid profiles and insulin sensitivity [23,24,25], and similar doses enhanced muscular strength and recovery in physically active populations [20,21,22]. Lower doses also exhibit biological activity, though their effects are generally less pronounced and often limited to mild hormonal or subjective outcomes [12,16]. Overall, the most robust clinical benefits are observed at daily doses of 500–600 mg of standardised extract over several weeks, highlighting the importance of dose optimisation in therapeutic and functional applications.

In view of the above, the aim of this study was to determine the effects of 8-week ashwagandha supplementation (600 mg/day) combined with HIIT on the lipid profile and selected parameters of energy metabolism in healthy young men. The study focused on analysing changes in blood concentrations of key metabolic regulatory hormones related to adipose/muscle tissues, such as adiponectin, asprosin and irisin, in response to the training and supplementation intervention. It was hypothesised that ashwagandha supplementation combined with HIIT would result in greater improvements in lipid profile and energy metabolism-regulating hormones compared to HIIT alone, providing an effective strategy for improving metabolic health and reducing the risk of cardiovascular disease and obesity.

## 2. Materials and Methods

### 2.1. Participants

Healthy men (students) who met specific inclusion criteria (not engaged in competitive sports, and not participating in any regular training programme for at least 12 months prior to enrolment, but attending practical classes included in their study curriculum) participated in the study. Exclusion criteria included participation in competitive sport, use of tobacco products, consumption of alcohol, use of performance-enhancing drugs, sedatives, anti-anxiety or sleep medications, history of orthopaedic trauma or surgery in the past 6 months, history of chronic disease, known intolerance to herbal supplements of similar composition, and use of herbal preparations or supplements containing antioxidant and anti-inflammatory substances in the past 3 months. Recruitment of participants was conducted among students of the Faculty of Physical Education and Health in Biała Podlaska. Prior to the experiment, participants were randomly assigned to two groups in a double-blind manner. Of the 41 students meeting the inclusion criteria, 20 were assigned to the Ashwagandha group and 21 to the Placebo group. The randomisation process was conducted using the R programme which is widely available at https://CRAN.R-project.org/package=randomizeR (accessed on 12 October 2025) [28]. Three participants (two in the Ashwagandha group and one in the Placebo group) dropped out of the study for personal reasons. Finally, 38 students (18 in the Ashwagandha group, 20 in the Placebo group) were included in the statistical analysis.

The study was conducted in accordance with the principles of the Declaration of Helsinki. All participants gave written consent to participate, and the study protocol was approved by the Local Ethical Committee of the Józef Piłsudski University of Physical Education in Warsaw (SKE-01-43/2022). During the study, participants were obliged to refrain from dietary modification.

### 2.2. Supplementation

Participants received supplementation in the form of two ashwagandha extract capsules daily (2 × 300 mg), for a period of eight weeks. Each capsule contained 300 mg of ashwagandha KSM-66 root extract, standardised to contain 5% withanolides, as determined by HPLC.

The placebo capsules, containing rice flour, microcrystalline cellulose, magnesium stearate and caramel as colour, were identical in appearance (size, shape and colour) and were manufactured by the same manufacturer. Participants in both the Ashwagandha and Placebo groups were required to take the capsules twice daily, after meals (breakfast and dinner). The supplements were dispensed to participants weekly in dark, opaque bottles. In both the study and control groups, no adverse effects were reported, and good tolerance of the ingested capsules was reported.

### 2.3. Nutritional Status

The dietary assessment was carried out using the method of ongoing recording. Participants were required to accurately record the liquids and meals consumed, considering weight units and economic measures. The recording included three days: two working days and one day off (study), both before the start of the experiment and after the end of the 8-week study period. To accurately determine the weight of food products, subjects were able to use the ilewazy.co.uk tool. Qualitative and quantitative analysis of the data was carried out using the Kcalmar.pro application, assessing the energy value and micro- and macronutrient content of the participants’ diets. The dietary analysis included an assessment of the intake of the basic macronutrients: protein, fats and carbohydrates, as well as dietary fibre and total calories of the diet. The reference values to which the data obtained were compared were calculated individually for each subject. Calculations of basal metabolism (PPM) were made using the Mifflin–St Jeor formula for men [29]:

PPM = 10 × body weight (kg) + 6.25 × height (cm) − 5 × age (years) + 5

and then this result was multiplied by the Physical Activity Level (PAL). The formula [30] was used to calculate the total energy requirement

(CPM): CPM = PPM × PAL

### 2.4. Training Protocol

Before and after the training period (at least 48 h after the last training session), the students underwent a graded exercise test (rowing ergometer Concept 2) to volitional fatigue (i.e., with a gradual increase in intensity until the subjects had to stop due to exhaustion, the GXT test). A detailed description of this test is described in a previous paper [30].

The eight-week HIIT was performed on a Concept 2 rowing ergometer (Morrisville, NC, USA) under the supervision of qualified instructors. The training programme consisted of 3 sessions per week, with at least 1 rest day between sessions. During the first two weeks (weeks 1–2), the training was designed to prepare participants for high-intensity exercise, so the load was 75–80% of the individual’s maximal power (Pmax), as measured during the GXT, and the training included 5 series of 1.5 min each, with 1.5 min active breaks at 70 W. In the following weeks, the load was increased to 85–95% Pmax (5–7 series of 1.5 min each). The HIIT programme was based on the work of Driller et al. [31] with modifications. In contrast, a detailed description of the training programme was presented in a previous paper [32]. Table 1 shows the scheme for conducting the training.

### 2.5. Body Composition

Body mass and selected body composition parameters were determined twice, i.e., before the start of the experiment (term 1) and after the end of the experiment, i.e., after 8 weeks of training and supplementation (term 2). Body weight (kg) and selected body composition parameters, fat mass (FM, %), muscle mass (involving skeletal muscle tissue, MM, %) and total body water (TBW, %), were obtained using a Tanita TBF 300P body composition analyser (Japan, China), which uses the electrical bioimpedance (BIA) method.

### 2.6. Blood Sampling and Diagnostics

Blood samples from the ulnar vein were taken on two dates: before the start of the experiment (term 1) and after the end of the experiment (term 2). At each term, the blood sample was taken three times: in the morning at 7:00 am (after an overnight fast, the “pre” point), at the end of the GXT (the “post” point) and 24 h after the end of the GXT (the “post 24 h” point). Blood samples were collected into a tube (2 mL) with anticoagulant (EDTA). Blood samples were taken by properly trained medical staff from the local hospital. Hormone determinations were performed using a Biotek reader together with an automatic scrubber (Bio Tek Instruments, Seattle, WA, USA) and dedicated software (Gen 5 Image+ v. 2.09). Determinations of lipid profile parameters were performed on an A15 automatic biochemical analyser (BioSystem S.A., Barcelona, Spain). Both analysers are on the equipment of the Department of Physiology and Biochemistry of the Faculty of Physical Education and Health in Biała Podlaska. The analyses were performed by a suitably trained analytical staff member of the department. The analyses were performed twice for each sample, and the mean values were calculated. For all biochemical parameters analysed, the mean intra-assay coefficient of variation (calculated for repeated samples) ranged between 4.1 and 7.8%. Table 2 summarises the parameters analysed, and the methods used for their determination.

### 2.7. Statistical Analysis

Statistical analyses were performed using Statistica software version 13.3 (StatSoft, Kraków, Poland). Assumptions regarding the normality of the distribution of all variables were verified using the Shapiro–Wilk test and analysis of quantile distribution plots. Homogeneity of variance was tested using Levene’s test. All results are presented as mean ± standard deviation (SD). A *p*-value of <0.05 was taken as the level of statistical significance. An unpaired Student’s t-test was used to assess differences between groups (Ashwagandha vs. Placebo) in anthropometric characteristics (age, height, etc.).

Analysis of body composition and diet was performed using two-factor analysis of variance (ANOVA) for repeated measures: 2 groups (Placebo, Ashwagandha) × 2 terms (pre-intervention term 1 and post-intervention term 2). A two-factor ANOVA with repeated measures was used to analyse lipid profile parameters and selected hormones: 2 groups × 2 terms (term 1 and term 2) × 3 time points (“pre”, “post”, “post 24 h”), with the data previously subjected to a logarithmic transformation (natural logarithm). For detailed comparisons (between and within groups), the Tukey post hoc test for unequal sample sizes was used. The sample size was calculated using GPower version 3.1 [33]. Assuming an effect size of f = 0.25, α = 0.05 and a test power of 0.8, the required sample size for a two-factor ANOVA with repeated measures was 34.

## 3. Results

The characteristics of the study subjects are shown in Table 3. In terms of the anthropometric indices analysed and BMI, there were no statistically significant differences between the groups (*p* > 0.05).

Analysis of the macronutrient content of the subjects’ diets (Table 4) showed that the subjects’ diets complied with the Human Nutrition Standards for the Polish Population and were 20% for protein, 30% for fat and 50% of daily energy requirements were covered by carbohydrates. The amount of fibre was compared to the recommendations for intake at the adequate level, which is 25 g [34]. Lower daily calorie intake was observed in the Ashwagandha and Placebo groups compared to the average reference values. These differences range from approximately 300–570 kcal per day. After the experiment, a decrease in calorie intake was noticed, especially in the ashwagandha supplement group. A lower than recommended protein intake of approximately 16–26 g was also observed in both groups. Respondents in the Ashwagandha group consumed lower amounts of this macronutrient, and these changes were observed during the post-intervention dietary assessment. In both groups, carbohydrate intake was lower than the reference values. In the Ashwagandha group, there was a statistically insignificant decrease in the second term, while in the placebo group there was a maintenance of the intake of this macronutrient. In the dietary assessment, fat intake was also analysed. It was observed that the subjects consumed lower than recommended amounts of fats by an average of 20–25 g per day. Another component that was analysed was fibre, whose values were close to or statistically non-significantly higher than the reference values. The study group showed a statistically insignificant decrease in fibre intake at the second study date, but the values still remained at the recommended intake.

Selected body composition parameters were analysed. Considering the available reference standards [35], the TBW and FM values are within the normal range. Analysis of the selected body composition parameters of the subjects (Table 5) showed statistically non-significant changes after 8 weeks of the study, both in the Ashwagandha group and in the Placebo group (*p* > 0.05 for main effect of group, term and group × term interaction).

Table 6 shows the values of lipid profile parameters studied in both groups. According to the recommendations given by the Polish Society of Laboratory Diagnostics and the Polish Lipidological Society, the values of the parameters studied did not exceed the accepted reference ranges [36]. In both groups, it was observed that the values of tChol, HDL-cholesterol and LDL-cholesterol parameters decreased statistically insignificantly after 8 weeks of the experiment. At the same time, a statistically insignificant increase was also observed for the TG parameter. Statistical analysis did not show for any of the lipid profile parameters analysed a statistically significant main effect of time (exercise test), term (training), or group (ashwagandha supplementation), or an interaction between these factors.

Table 7 shows the changes in blood values of selected hormones. Analysis of the changes in adiponectin concentration induced by 8-week HIIT showed a significant interaction of time and term (*p* < 0.05). At the “pre” point, there was a decrease in the values of this parameter in both groups, except that the change in the Ashwagandha group was statistically significant (*p* < 0.05).

For the analysis of asprosin concentration (Table 7) in term 2, compared to term 1, there was a decrease in the value of this parameter within both groups at the “pre” and “post” points and an increase at the “post 24 h” point. However, all these changes were statistically insignificant (no significant main effects or interaction, *p* > 0.05).

Analysis of irisin concentration (Table 7) showed that 8-week HIIT contributed to changes in this parameter (main effect of time, *p* < 0.001 and interaction of time and term, *p* < 0.0001). In both groups in term 2, an increase in irisin concentration was noted at the “post” point, compared to the “pre” point, except that in the Placebo group this change was statistically significant (*p* < 0.01). Also in term 2, statistically significant increase in irisin was noted at the “post 24 h” point within both groups (in Ashwagandha group: “post 24 h” vs. “post” and “pre” points, *p* < 0.0001; in Placebo group: “post 24 h” vs. “pre” points, *p* < 0.0001).

## 4. Discussion

The aim of our study was to evaluate the effect of ashwagandha supplementation in combination with HIIT on metabolic health indicators, with a particular focus on cardiovascular disease risk. The main finding is that even in young, healthy men, 8 weeks of HIIT can improve certain parameters related to energy metabolism, but ashwagandha does not appear to offer additional benefits in this regard.

It is well known that excessive adipose tissue promotes chronic low-grade inflammation (by secreting numerous adipokines and pro-inflammatory cytokines), which further compounds the adverse effects on human health (insulin resistance, dyslipidaemia, hypertension and an increased risk of developing cardiovascular disease). This is why undertaking physical activity is so important not only as an elimination of the causes of disorders that have already occurred, but above all as a preventive factor.

In recent years, several clinical studies have confirmed the beneficial effects of high-intensity interval training (HIIT) on subjects’ body composition, including primarily fat reduction and increases in lean body mass, including muscle. An analysis of the scientific literature from recent years showed that the authors mainly focused on studying the effects of HIIT in overweight subjects. Tsirigkakis et al. showed that eight-week HIIT protocols lead to a significant reduction in visceral fat [37]. Similar observations were reported by Poon et al. who, in a study involving obese middle-aged men, found that both HIIT and moderate-intensity continuous training resulted in comparable decreases in fat mass and waist circumference [38]. In contrast, D’Alleva et al. documented that a 12-week HIIT programme improved body composition to a degree like combined training, while improving fat metabolism [39]. The results obtained in the experiment described in this article indicate that 8-week HIIT does not induce changes in body fat in subjects with normal body weight. This is surprising given the dietary analysis, which showed a tendency in both groups toward a decrease (albeit insignificant) in daily calorie intake at term 2 compared with term 1. On the other hand, it should be emphasised that the method of assessing diet based on information provided by participants may be a limitation of the study, as self-assessment can be biased.

The lipid profile, including the concentration of total cholesterol, HDL and LDL fractions and triglycerides, is one of the basic laboratory tests for assessing the body’s lipid metabolism. These parameters play a key role in the diagnosis, prevention and monitoring of cardiovascular diseases, which are among the leading causes of death worldwide. Findings from the past five years suggest that high-intensity interval training may have a beneficial effect on lipid profile in men, especially in overweight and obese populations [40,41,42,43]. The experiment described in this article showed a reduction (although not significant) in tChol, LDL-cholesterol after 8 weeks of training, which agrees with the results of other researchers. The trend towards a decrease in tChol and LDL-cholesterol after 8-week HIIT (i.e., a decrease at term 2 vs. term 1) observed in both groups was true at all time points (i.e., resting before the exercise test, at the end of the exercise test and within 24 h after exercise). On the other hand, this decrease also applied to HDL-cholesterol, which is difficult to interpret. It should be noted, however, that the studies described in this article concern young people with relatively high baseline HDL-cholesterol levels (70–80 mg/dL). It is worth noting at this point that the articles by other authors described above show results that are partly inconsistent: many studies report an improvement in TG and tChol/HDL-cholesterol ratio or an increase in HDL-cholesterol, but the effect depends on the length of the intervention, the volume of training, the calorie content of the daily diet and the characteristics of the subjects. In addition, Zhu et al. pointed out that HIIT may not only modify lipoprotein concentrations, but also improve lipoprotein function, which is of particular importance in the context of cardiovascular disease prevention [9].

Although the results found in the scientific literature are not conclusive, nevertheless, these data indicate that HIIT may be a promising strategy for improving the lipid profile in men, although the effects may vary depending on the characteristics of the study population, the training protocol used or the diet.

In addition to its role as a storehouse of high-energy substances, adipose tissue is an active endocrine organ, producing factors that regulate appetite, energy balance and inflammatory processes, including adiponectin [44]. Adiponectin is a key cytokine secreted by adipose tissue that plays a fundamental role in the regulation of insulin sensitivity [44,45,46], glucose [45,47] and fatty acid metabolism [48,49], as well as in anti-inflammatory and anti-atherosclerotic processes. Changes in its concentration are strongly associated with insulin resistance [44,45,46], risk of type 2 diabetes [50,51] and cardiovascular disease. Regular exercise plays an important role in modulating adiponectin levels in humans, with the effect depending on the type of intervention, its duration and changes in body weight. Mallardo et al. observed that a single exhaustive exercise in active men causes a rapid and sustained increase in adiponectin levels, which may indicate mechanisms of an acute adipocyte response independent of weight reduction [52]. Similarly, analysis of the results of the experiment described in this article showed that a single intense exercise (GXT) induced an increase in adiponectin levels (affecting both groups at term 2), although these changes were not statistically significant (*p* > 0.05). In contrast, in the experiment reported in this article, 8-week training induced a decrease in pre-exercise (resting) serum levels of this hormone (with term × time interaction; *p* < 0.05). A post hoc analysis showed that a significant decrease in pre-exercise adiponectin following 8-week HIIT (i.e., a decrease in values at term 2 vs. term 1) affected the Ashwagandha group. The data obtained are not clear and require further study but may be related to the lack of significant changes in body weight and body fat. A similar analysis was presented by Ouerghi et al. who studied an 8-week HIIT intervention that had no effect on adiponectin levels which was associated with no decrease in subjects’ body weight [53]. In conclusion, one-off intense exercise can increase adiponectin levels, while the effects of long-term physical activity (including physical training) appear to be strongly modulated by the nature of the training protocol, its duration and changes in body fat.

Another hormone secreted mainly by white adipose tissue is asprosin [54], which may control appetite and stimulate the release of glucose from the liver, especially during fasting [55]. Elevated levels of asprosin are observed in people with insulin resistance, obesity and type 2 diabetes, suggesting a role for this hormone in the development of these conditions [55,56]. In recent years, increasing evidence has accumulated that exercise lowers blood asprosin concentrations in humans, with the effect depending on the nature (acute vs. chronic), intensity and metabolic status of the subjects. Ceylan et al. observed that a single session of moderate to high-intensity exercise can lead to a short-term decrease in asprosin in lean and obese individuals, although, as they point out, the response is sometimes dependent on the BMI of the participants [57].

Meta-analytical and synthetic reviews including randomised controlled trials confirm a general trend of decreasing asprosin concentrations after exercise training programmes (different types: aerobic training, HIIT) in overweight and obese subjects, although the heterogeneity of protocols and populations explains the variation in effects between studies [58]. Analysing the impact of the 8-week experiment described in this article, a non-significant decrease in asprosin concentrations was noted in both groups (term 2 vs. term 1; this refers to pre- and post-exercise levels). The lack of significance may be due to large interindividual differences in the hormonal response to physical exercise (considering the SD values in Table 7), as well as the fact that our study included healthy men with normal body weight and body fat content. Therefore, the data suggest that long-term exercise exposures can reduce asprosin concentration. It is speculated that the mechanisms responsible for this phenomenon probably include a reduction in fat mass and an improvement in insulin sensitivity—but further well-designed studies in humans (different BMI groups, standardisation of intake time and exercise types) are needed to precisely characterise the timing and magnitude of the effect [58,59,60].

In recent years, numerous studies have indicated that both single, high-intensity physical activity and long-term training programmes can significantly increase irisin, a hormone released from muscle tissue cells, although the response is dependent on the type of exercise, its intensity and the metabolic status of the participants. In the experiment reported in this article, 8 weeks of HIIT intervention in both groups resulted in a significant increase in irisin concentration after the graded GXT test and during the 24 h recovery period (main effect of time, *p* < 0.001 and term × time interaction, *p* < 0.0001). Similar results were obtained by Hasanah et al. in obese women and showed that all forms of combined exercise (aerobic and strength), especially at high intensity, significantly increased irisin levels [61]. Also, Küçük et al. investigating a HIIT intervention in male football referees observed a 2% increase in irisin after a single session and up to 106% after 12 weeks of training, confirming the cumulative effect of this type of activity [62].

A comparison of training types by Adilakshmi et al. showed that eight weeks of high-intensity resistance training raised irisin levels significantly more than endurance training [63]. Systematic reviews and meta-analyses indicate that exercise generally increases irisin concentrations, with HIIT being particularly effective in this regard, especially in overweight individuals [64,65]. The results obtained in the experiment described in this article indicate that HIIT may have a beneficial effect on energy metabolism, since irisin can exert a cytoprotective effect, reducing oxidative stress and inflammation, by stimulating, among others, the “browning” process [66]. In addition, irisin has beneficial effects on bone mineral density muscle mass, and regular physical activity maintains its normal levels, protecting against osteoporosis [67,68], sarcopenia and atherosclerosis [69,70]. Finally, our results confirm the validity of using HIIT as a preventive intervention in young, healthy men with normal body weight.

One of the tasks of the present experiment was to determine whether ashwagandha supplementation could enhance the effectiveness of HIIT programme in improving parameters related to energy metabolism. The potential of ashwagandha to exert beneficial effect on metabolic health may result from antioxidant and anti-inflammatory properties of ashwagandha’s active ingredients [27]. However, in our study, no effect of ashwagandha extract was observed for most of the parameters studied (*p* > 0.05 for main effect of group or interactions of group with time/term). Our results are difficult to compare due to the lack of studies in the available literature on ashwagandha supplementation in healthy individuals undergoing HIIT, as well as none of the studies on ashwagandha supplementation assessed the hormonal indicators of energy metabolism presented in this article.

In recent years, an increasing number of studies have indicated that *Withania somnifera* (ashwagandha) supplementation can significantly support exercise adaptation in both healthy untrained individuals and athletes [71,72,73]. A 2024 randomised controlled trial showed that ashwagandha supplementation (500 mg/day for 60 days) increased handgrip strength, muscle mass and physical performance, and positively modulated inflammatory markers compared to placebo [73]. Interestingly, however, these effects were observed without any controlled physical training, and participants were even asked to refrain from unaccustomed strenuous physical activity throughout the duration of the study [73]. It should also be noted that the BMI of some study participants exceeded normal values (it ranged 18–30 kg/m^2^). Similar results were obtained in untrained men undergoing an 8-week resistance training programme, where supplementation with ashwagandha (600 mg/day) significantly improved gains in both muscle strength and lean body mass, as well as a decrease in body fat percentage [74]. Analysing the results of the study presented in this article, no significant effect of 8-week supplementation with ashwagandha (600 mg/day) on body composition was observed. There was also no effect of supplementation on changes in physical performance parameters (VO_2max_, exercise time to exhaustion, maximal power, threshold power) beyond those observed under training alone (results published in [30]). Undoubtedly, the response of the body is dependent on the type of training effort, its intensity and the health status of the participants, as well as the level of physical activity. In contrast to the studies by other authors described above [73,74], the study described in this article involved physically active men (physical education students). It cannot be excluded that a longer period of ashwagandha supplementation may be needed to exert benefits in healthy population engaged in intense training regimen. On the other hand, Ziegenfuss et al., using a longer period of ashwagandha supplementation (12 weeks, 500 mg daily) in combination with strength training in recreationally active men, also reported no significant influence of ashwagandha on body composition (body fat percentage, lean mass or fat mass), although some favourable improvements in distribution of body mass and muscle strength were reported as a result of supplementation [22]. A strength of the research described in this article is undoubtedly that the training protocol was fully controlled and supervised, as opposite to other studies [22,74].

As with body composition, ashwagandha supplementation did not affect the lipid profile in the study described in this article, which is consistent with other studies in which ashwagandha was administered to healthy individuals engaged in strength training [22]. To date, the beneficial effect of ashwagandha on the lipid profile has been observed primarily in subjects with various types of medical conditions [23,25]. Taking together, the results described in this article are unique since, to our knowledge, the present study is the first to evaluate ashwagandha supplementation combined with HIIT on metabolic health parameters. On the other hand, our results regard healthy males only, that may be considered as the limitations of that study and therefore need to be verified in further studies across different training protocols, doses administered and study groups. Further research is warranted to address all the issues mentioned above.

## 5. Conclusions

Based on the results, it can be concluded that the 8-week HIIT programme has a significant effect on selected hormones related to adipose (adiponectin) and muscle (irisin) tissues. However, ashwagandha supplementation (600 mg/day) in healthy men subjected to 8-week HIIT does not affect health status parameters (body composition, lipid profile), as well as selected hormones related to energy metabolism). Further research is needed on other doses of ashwagandha and/or supplementation duration in other populations (older women and men, overweight/obese individuals).

## Figures and Tables

**Table 1:** HIIT programme.

Week 1st–2nd	Week 3rd–4th	Week 5th–6th	Week 7th–8th
5 series of 1.5 min.	5 series of 1.5 min.	6 series of 1.5 min.	7 series of 1.5 min.
75–80% Pmax 70 W 1.5 min. breaks	85% Pmax 70 W 1.5 min. breaks	90% Pmax 70 W 1.5 min. breaks	95% Pmax 70 W 1.5 min. breaks

Pmax—individual’s maximal power.

**Table 2:** Summary of analysed parameters.

Parameter	Manufacturer	Method	Sensitivity (Detection Limit)
tChol	Alpha Diagnostics (Warsaw, Poland)	enzymatic colorimetric	4 mg/dL
HDL-chol	Alpha Diagnostics (Warsaw, Poland)	enzymatic colorimetric	1.06 mg/dL
LDL-chol	Spinreact (Girona, Spain)	enzymatic colorimetric	1 mm/dL
TG	Alpha Diagnostics (Warsaw, Poland)	enzymatic colorimetric	2 mg/dL
Adiponectin	Biorbyt (Cambridge, UK)	ELISA	0.11 mg/L
Asprosin	Biorbyt (Cambridge, UK)	ELISA	0.15 ng/mL
Irisin	Biorbyt (Cambridge, UK)	ELISA	0.938 ng/mL

**Table 3:** Baseline characteristics of the study participants (mean ± SD).

Variable	Ashwagandha (*n* = 18)	Placebo (*n* = 20)
Age (years)	20.3 ± 0.8	20.8 ± 1.8
Body height (cm)	181.9 ± 6.3	182.8 ± 7.6
Body weight (kg)	81.2 ± 9.6	79.2 ± 9.5
BMI (kg/m^2^)	24.6 ± 2.7	23.7 ± 1.8

No inter-group differences were found at *p* < 0.05 (unpaired Student *t*-test).

**Table 4:** Quantitative characteristics of selected components of the daily diet in the study participants (mean ± SD).

Component Time	Ashwagandha (*n* = 18)	Placebo (*n* = 20)
Protein (g)		
“term 1”	131.5 ± 49.4	129.1 ± 42.0
“term 2”	121.0 ± 44.8	128.4 ± 41.4
Carbohydrates (g)		
“term 1”	334.4 ± 128.7	333.2 ± 112.8
“term 2”	287.3 ± 119.1	333.2 ± 145.7
Fat (g)		
“term 1”	77.0 ± 34.6	74.7 ± 35.4
“term 2”	73.3 ± 31.3	73.5 ± 35.0
Fibre (g)		
“term 1”	26.7 ± 12.9	27.8 ± 12.1
“term 2”	23.2 ± 13.0	26.0 ± 11.5
Caloric intake (kcal)		
“term 1”	2565.9 ± 825.7	2518.78 ± 779.1
“term 2”	2377.1 ± 819.7	2472.8 ± 828.4

“term 1”—results obtained before the experiment; “term 2”—results obtained after the experiment (after 8 weeks). No main effect of group, term and group × term interaction was found (*p* > 0.05).

**Table 5:** Body weight and selected body composition components in the study participants (mean ± SD).

Component Time	Ashwagandha (*n* = 18)	Placebo (*n* = 20)
Body weight (kg)		
“term 1”	81.2 ± 9.6	79.2 ± 9.5
“term 2”	81.1 ± 9.4	79.3 ± 11.2
TBW (%)		
“term 1”	61.8 ± 3.0	62.4 ± 3.2
“term 2”	62.3 ± 3.0	62.5 ± 3.5
MM (%)		
“term 1”	80.6 ± 4.0	81.4 ± 4.1
“term 2”	81.1 ± 4.1	81.5 ± 4.5
FM (%)		
“term 1”	15.6 ± 4.1	14.6 ± 4.9
“term 2”	15.0 ± 4.5	14.8 ± 4.3

“term 1”—results obtained before the experiment; “term 2”—results obtained after the experiment (after 8 weeks); TBW—total body water amount; MM—muscle mass; FM—fat mass. No main effect of group, term and group × term interaction was found (*p* > 0.05).

**Table 6:** Results of the lipid profile in the study participants (mean ± SD).

Component Time	Ashwagandha (*n* = 18) “Term 1”	Ashwagandha (*n* = 18) “Term 2”	Placebo (*n* = 20) “Term 1”	Placebo (*n* = 20) “Term 2”
tChol (mg/dL)				
“pre”	180.7 ± 18.0	168.5 ± 22.3	168.8 ± 23.7	161.7 ± 25.4
“post”	204.8 ± 36.7	190.2 ± 30.3	189.6 ± 25.3	181. 2 ± 25.8
“post 24 h”	186.5 ± 23.1	177.0 ± 26.0	175.1 ± 27.3	172.7 ± 25.2
HDL (mg/dL)				
“pre”	78.6 ± 15.0	71.6 ± 11.8	78.7 ± 17.1	71.4 ± 13.8
“post”	83.3 ± 14.7	81.6 ± 16.3	80.8 ± 18.1	81.1 ± 17.4
“post 24 h”	80.8 ± 14.0	75.3 ± 14.5	78.1 ± 15.6	77.6 ± 15.6
LDL (mg/dL)				
“pre”	132.3 ± 24.1	128.5 ± 29.8	120.1 ± 31.0	117.3 ± 31.7
“post”	144.4 ± 35.9	135.0 ± 31.9	126.7 ± 33.1	123.8 ± 31.8
“post 24 h”	137.1 ± 30.4	129.0 ± 29.2	126.0 ± 33.4	119.8 ± 27.7
TG (mg/dL)				
“pre”	104.6 ± 48.1	110.0 ± 43.1	107.5 ± 53.1	118.9 ± 50.4
“post”	144.0 ± 63.5	147.6 ± 90.2	162.6 ± 79.4	155.3 ± 64.7
“post 24 h”	94.7 ± 32.2	123.0 ± 95.1	100.3 ± 37.6	99.2 ± 37.2

“term 1”—results obtained before the experiment; “term 2”—results obtained after the experiment (after 8 weeks); “pre”—results obtained before graded exercise test; “post”—results obtained post graded exercise test; “post 24 h”—results obtained 24 h after graded exercise test. No main effect of group, term, time, as well as interaction were found (*p* > 0.05).

**Table 7:** Results of examined hormone profile tests in the study participants (mean ± SD).

Component Time	Ashwagandha (*n* = 18) “Term 1”	Ashwagandha (*n* = 18) “Term 2”	Placebo (*n* = 20) “Term 1”	Placebo (*n* = 20) “Term 2”
Adiponectin (mg/L)				
“pre”	6.29 ± 2.9	4.36 ± 1.9 ^#^	5.76 ± 3.5	4.61 ± 1.9
“post”	4.83 ± 3.5	5.26 ± 3.9	4.07 ± 3.0	5.46 ± 3.4
“post 24 h”	4.69 ± 1.9	4.62 ± 2.1	5.41 ± 2.3	5.14 ± 2.5
Asprosin (ng/mL)				
“pre”	3.79 ± 3.8	2.85 ± 4.0	3.51 ± 3.3	3.14 ± 3.8
“post”	4.56 ± 4.3	3.10 ± 2.3	4.43 ± 4.9	4.12 ± 6.9
“post 24 h”	3.94 ± 4.2	5.20 ± 9.1	3.94 ± 4.7	4.33 ± 5.0
Irisin (ng/mL)				
“pre”	89.32 ± 36.70	89.62 ± 37.40	80.99 ± 35.20	79.89 ± 37.40
“post”	87.07 ± 38.50	95.08 ± 46.40	77.86 ± 34.60	90.46 ± 37.30 ^††^
“post 24 h”	85.73 ± 37.90	106.48 ± 42.20 ***	78.61 ± 36.90	95.43 ± 41.50 ^†††^

“term 1”—results obtained before the experiment; “term 2”—results obtained after the experiment (after 8 weeks); “pre”—results obtained before graded exercise test; “post”—results obtained post graded exercise test; “post 24 h”—results obtained 24 h after graded exercise test. Significant main effects for adiponectin: term × time interaction (*p* < 0.05) ^#^—significant post hoc differences in adiponectin (term 2 vs. term 1 in “pre” value, *p* < 0.05, within the same group: ashwagandha). Significant main effects for irisin: time (*p* < 0.001), term × time interaction (*p* < 0.0001) ***—significant post hoc differences in irisin (“post 24 h” vs. “post” and “pre” values, *p* < 0.0001, in term 2, within the same group: ashwagandha) ^††, †††^—significant post hoc differences in irisin (“post” vs. “pre” value, *p* < 0.01 and “post 24 h” vs. “pre” value, *p* < 0.0001, respectively; in term 2, within the same group: placebo).

## Data Availability

The data presented in this study are available on request from the corresponding author due to legal restriction (Polish law relating to the protection of personal data).

## Abbreviations

The following abbreviations are used in this manuscript:

PPM	Basal metabolism
HIIT	High-intensity interval training
PAL	Physical Activity Level
CPM	Total energy requirements
TBW	Total body water
MM	Muscle mass
FM	Fat mass
BIA	Body Impedance Analysis
tChol	Total cholesterol
HDL	High-density lipoprotein
LDL	Low-density lipoprotein
VLDL	Very low-density lipoprotein
FFA	Free fat acids
HPLC	High Performance Liquid Chromatography
ET	Exercise training
